# Editorial: Long COVID: pathogenesis, diagnosis and clinical management

**DOI:** 10.3389/fmed.2025.1615692

**Published:** 2025-05-16

**Authors:** Arch G. Mainous, Shisan Bao, Michel Goldman

**Affiliations:** ^1^Department of Community Health and Family Medicine, University of Florida, Gainesville, FL, United States; ^2^Department of Health Services Research, Management, and Policy, University of Florida, Gainesville, FL, United States; ^3^Center for Laboratory and Simulation Training, School of Public Health, Center for Evidence-Based Medicine, Gansu University of Chinese Medicine, Lanzhou, Gansu, China; ^4^Institute for Interdisciplinary Innovation in Healthcare (I3H), Université Libre de Bruxelles, Brussels, Belgium

**Keywords:** COVID, Long COVID, clinical management, diagnosis, mortality

Five years have passed since Coronavirus disease 2019 (COVID-19) rapidly spread worldwide, causing the biggest public health crisis in 21^st^ Century (1, 2). While acute severe damages caused by the SARS-CoV-2 virus COVID-19 were eventually controlled with vaccines, public health measures and surveillance systems, the long-term effects of the infection are yet to be fully understood. The medical community now has a new battle to confront: long COVID, also known as Post COVID Condition or Post Acute Sequelae of COVID-19. The public health impact of Long COVID is huge with millions of people affected and major economic and societal consequences. Indeed, Long COVID is estimated to affect more than 400 million people worldwide with the estimated prevalence of long COVID in the US adult population being 7.5% (3, 4). Long COVID has been linked to an increase in both hospitalizations and death from a variety of causes (5, 6). Less severe outcomes associated with long COVID are significant activity limitations and even increased work days missed due to illness (7, 8).

From a clinical standpoint, long COVID is not a homogeneous disease. It should be viewed as a multisystemic syndrome which persists for at least 3 months after acute COVID-19 (1, 2). It might cause a wide range of symptoms so that its differentiation from other conditions is very challenging, as further underlined in several articles of this series which provide additional insight into different aspects of Long COVID (9).

The influence of smoking and obesity on risk of hospitalization was addressed by Fernández-Pedruelo et al. In an analysis of medical records of a sample of patients diagnosed with COVID-19 in Spain, the results showed that patients with obesity had a significantly higher risk of post acute sequelae, namely memory disorders, from COVID-19. Importantly, smoking was not directly related as a long COVID complication. Neither of these factors was associated with an increased risk of hospitalization. This study points to the importance of different long COVID complications and factors that may increase the risk for one type of complication but not another.

The prevalence of long COVID and corresponding risk factors was investigated in several studies (Aldhawyan et al.; Saloma et al.). The value of these studies showed that long COVID is a world wide problem. As we gain more knowledge about long COVID it is important to keep in mind that to appropriately address long COVID we need to adopt a global viewpoint. Saloma et al. provided new information on the impact of new variant, Theta, of SARS-CoV-2 on the prevalence of long COVID. Ruiyin et al. focused on long COVID from the Omicron variant. These studies highlight the influence of different SARS-CoV-2 variants in different parts of the world and their resulting long COVID. The need for long-term monitoring of long COVID is apparent as well as the impact on human health and the need for our health systems to adopt policy response strategies.

Similar to a need for a global view on prevalence of long COVID, several studies focused on the more extreme complication of long COVID, mortality (Grippo et al.; Won et al.). Studies from Italy and South Korea showed that long COVID complications can be very severe. Although this effect was shown in the US it is important to add these additional international studies to our body of knowledge (5). Strategies to identify the population at risk of severe long-term consequences of SARS-CoV-2 infection and interventions aimed at reducing this risk must be developed.

Several review articles were also included in this Research Topic (Ranque and Cogan; Dietz and Brondstater). These reviews bring to the forefront current strategies for managing and preventing long COVID. A particular value of these reviews is that they show what we know and what we don't know. Further, they point to promising new strategies and underutilized modalities.

A study that focused on a much different aspect of our toolbox for long COVID was the study by Sperl et al.. This study on long COVID focused on the psychometrics involved in the translation and adaption of the COVID-19 Yorkshire Rehabilitation Scale for a German patient population. Activity limitations and complications like dyspnea are not uncommon in long COVID. As might be expected there were some modifications both in terms of translation and cultural references needed to make the English version work for German populations. This study reinforces that long COVID is a global problem and we need to pull information learned in one country to help other countries more successfully deal with the problem.

Finally, this Research Topic of articles included a series of case reports on the commonly reported long COVID complication of fatigue (Morelli-Zaher et al.). Patients with excessive daytime sleepiness was assessed for objective central hypersomnia. This series of case reports shows that methylphenidate was a promising treatment for these patients. This leads to the conclusion that the long COVID complication of central hypersomnia needs to be included in the physicians' differential diagnosis because it may be a treatable condition.

In conclusion, this group of articles points to the global impact of long COVID as well as the varied complications from the condition. As the focus on COVID-19 wanes in the popular press, it is incumbent upon us all to ensure that long COVID is handled as a major public health priority requiring additional research on its pathogenesis and treatment.
